# Supplementary material for: Patient Satisfaction with a Dedicated Infusion Pump for Subcutaneous Treprostinil to Treat Pulmonary Arterial Hypertension
Source: J Pers Med. 2023 Feb 26;13(3):423. doi: 10.3390/jpm13030423 (PMC10058864; doi:10.3390/jpm13030423)
Supplement: Supplementary file 1 [file jpm-13-00423-s001.zip › jpm-2219900-supplementary.pdf]

SUPPLEMENTARY MATERIAL

Supplementary Table S1. Available pumps for subcutaneous treprostinil administration in Poland. Characteristics according to the manufacturer specifications.

	**I-JET®**	**Micro sc Infusion Pump®**	**Canè Crono SC®**
**Size [mm]**	45.6 x 81.1 x 21.8	58 x 88 x 20	49 x 138 x 29 (including protective wings)
**Weight [g]**	70 g without battery	105 g (with battery)	127 g (including battery and protective wings)
**Display**	Color OLED	Color LCD	Monochromatic LCD
**Infusion rate range [ml/h]**	0.001 to 0.125	0.001 to 0.350	0.005 to 2.000
**Infusion rate increment [ml/h]**	0.001	0.001	0.001
**Units**	mL/h	µL/h	µL /h
**Delivery accuracy**	±5%	±5%	±2%
**Cartridge size**	3 mL	3 mL	10 and 20 mL
**Dust protection**	IP 5X	IP 5X	IP4X
**Moisture protection**	IP X8	IPX7	IPX2
**Battery type**	AAA	AAA	123A (3 V)
**Manufacturer**	Everaid Co. Ltd.<br>Goyang City, Gyeonggi-Do, Korea	Wuxi Apex Medical Co.<br>Jiangsu, China	Canè SpA<br>Rivoli (TO) - Italy
**EU Authorised representative**	Ferrer<br>Barcelona, Spain	Sci-Pharm (Member of the AOP Health Group)<br>Luxembourg	Canè SpA<br>Rivoli (TO) - Italy
**CE Mark Notified Body**	1639	0344	0476
CE: from the French, Conformité Européene, EU: Europe, g: gram, h: hours, IP: ingress protection, LCD: liquid-crystal display, µl: microlitre, mm: millimetre, ml: millilitre, OLED: organic light emitting diode			

Supplementary Figure S1. Patient Satisfaction Questionnaire

My JET - user satisfaction with Everaid I-Jet pump for subcutaneous Remoduline application

Table 1

1. How many years of experience do you have using a pump for SC administration?	Less than 1 year	1 to 3	3 to 5	5 to 8	more than 8 years
	○	○	○	○	○
2. What age group do you belong to (years)?	below 20	20 - 39	40 - 59	60 – 79	80 +
	○	○	○	○	○
3. Which gender you are?		Female		Male	
		○		○	

Table 2

Legend: 1 - very dissatisfied 2 - dissatisfied 3 - neutral 4 – likely satisfied 5 - very satisfied

Most liked technical features:	1 ☹☹	2 ☹	3	4 ☺	5 ☺☺
4. Do you like the style & design of the i-JET pump?	○	○	○	○	○
5. How would you rate the size & weight of the i-JET pump?	○	○	○	○	○
6. Are you satisfied with the legibility of the LCD color display?	○	○	○	○	○

Most liked technical features:	1 ☹☹	2 ☹	3	4 ☺	5 ☺☺
7. How would you rate the content shown on the display?	○	○	○	○	○
8. Are you satisfied with the use of the keyboard?	○	○	○	○	○
9. How would you rate the access to the battery compartment?	○	○	○	○	○
10. The pump is water resistant, how would you rate the importance of this?	○	○	○	○	○

Table 3

Set up of the pump/Usability:	1 ☹☹	2 ☹	3	4 ☺	5 ☺☺
11. How would you rate the clarity of information in the user interface?	○	○	○	○	○
12. How satisfied are you with the easy access to the settings menu?	○	○	○	○	○
13. How would you rate the intuitive software design of the user interface?	○	○	○	○	○
14. The flow rate can be set in 0.001 ml steps, how would you rate the importance of this?	○	○	○	○	○

Table 4:

Daily use/Ease of use/Simplicity:	1 ☹☹	2 ☹	3	4 ☺	5 ☺☺
15. How would you rate the access to the syringe compartment?	○	○	○	○	○
16. How would you rate the handling with the i-Life syringe?	○	○	○	○	○

Daily use/Ease of use/Simplicity:	1 ☹☹	2 ☹	3	4 ☺	5 ☺☺
17. Are you satisfied with the battery change process?	o	o	o	o	o
18. How would you rate the 'History' function, having opportunity to check/monitor therapy?	o	o	o	o	o
19. How would you rate the 'reminders' of the pump related to your personal safety?	o	o	o	o	o
20. How many occlusion alarms have you had in the past <u>30 days</u>?	NR#:				
	Yes	No			
21. Do you think that the administration with the new device is safe?	o	o			

Table 5

Training and instructions:	1 ☹☹	2 ☹	3	4 ☺	5 ☺☺
22. How satisfied are you with the quality of the i-JET instructions?	o	o	o	o	o
23. How would you rate our additional instructions and training materials?	o	o	o	o	o
24. How would you rate the comprehensibility of the operating instructions manual?	o	o	o	o	o
25. How would you overall rate the professionalism of our/i-JET service?	o	o	o	o	o

Table 6

Legend: 1 – not recommend 2 – rarely recommend 3 - neutral 4 – likely 5 – most likely recommend

Additional services:	1 ☹☹	2 ☹	3	4 ☺	5 ☺☺
26. How would you rate the usability of the ancillaries?	o	o	o	o	o
27. Are you satisfied with the available ancillaries (Quick set)?	o	o	o	o	o
28. Are you satisfied with the availability and delivery of our ancillaries?	o	o	o	o	o
29. The battery life is up to 2 - 3 weeks. Would you be satisfied with a shorter battery lifetime for additional technical features, like a WEB based service, etc.?	o	o	o	o	o
30. If you could change one thing about the pump, what would it be, please add your comment:					
31. How likely would you recommend this product to someone else?	o	o	o	o	o
32. How likely would you recommend Ferrer& the services to someone else?	o	o	o	o	o

Legend: 1 - very dissatisfied 2 - dissatisfied 3 - neutral 4 – likely satisfied 5 - very satisfied

33. Please rate <u>your overall satisfaction with the i-JET pump</u>/device (Max. 5 points- very satisfied): 1 ☹☹ 2 ☹ 3 - neutral 4 ☺ 5 ☺☺

Supplementary Figure S2. Patient Benefits & Education Questionnaire

Patient identifier: [ ] Date: [ ]

Patient Benefits & Education Questionnaire - i-JET pump

1. When did you started i-JET pump therapy? Date (year/month)______________________________

2. What are your expectations of i-JET pump therapy?

____

3. Would you consider yourself in good control through subcutaneous administration of Remodulin (treprostinil)?

O Yes O No

4. Were there any technical issue with your pump in the last 1 month which required you to go to the hospital and attend on an extra visit (not previously scheduled)? O Yes O No

If yes, describe what kind of issue it was, how was it managed?______________________________

5. Were there any error in the last month (last 4 weeks) which led to I-JET pump change? O Yes O No

---

Benefits of i-JET and Education

6. What are the benefits of i-JET pump therapy according to you?

____

7. Will you prefer using i-JET pump for treprostinil therapy in the future? O Yes O No

8. How easy this was for you to learn how to use your subcutaneous i-JET pump? (Maximum 5 points)

1 – very difficult 2 - somewhat difficult 3 - neutral 4 – easy to learn 5 – it is very easy to learn

9. Do you think it would be necessary to re-train you how to use your i-JET pump? O Yes O No

if yes, how frequently ?____

10. Did you receive appropriate help/training when you first started to use i-JET pump? O Yes O No

11. How satisfied are you with your i-JET pump? Please rate. Device satisfaction (Maximum points: 5)

1 - very dissatisfied 2 - dissatisfied 3 - neutral 4 – likely satisfied 5 - very satisfied

12. Please evaluate how easy it was to learn how to use the infusion set (Quick set) belonging to your pump. Infusion set evaluation (Maximum points: 5)

1 – very difficult 2 - somewhat difficult 3 - neutral 4 – easy to learn 5 – it is very easy to learn

Habits/Daily life with i-JET

13. How often do you change your infusion site? Please underline:

- not frequently – every 3 -4 weeks
- biweekly – after 2 weeks
- weekly

14. Please indicate which is your injection site preferance: O Abdomen O Thighs O Arms O Upper buttock

15. Did your life positively changed with i-JET pump therapy? O Yes O No O No impact at all

16. Is your pump hindering you in any activities of your life?

____

____

17. Do you disconnect the device/pump during shower? O Yes O No

18. In case of showering what is your current practise?

____

19. Overall do you find easy/convenient the device (i-JET pump) to live with? O Yes O No

Supplementary Figure S3. Nurse questionnaire

1. How long have you practiced PAH nursing/helped PAH patients as technician?
2. What is your age?
3. Is this the first time you have used an I-Jet pump?
4. Do you have experience with CADD MS3 or Apex pumps as well?
5. Does the I-Jet pump display show all the information that you think is necessary for patients?
6. Based upon your experience would you recommend/prefer other display options?
7. Is there any technical feature that you would want improved?
8. Please indicate if you agree with the statement below.
    a. I-Jet pumps improve patient quality of life
    b. I-Jet pump operation is user-friendly and easily learnt
    c. The presence of embedded alerts/alarms prevents drug administration error(s)
9. Nursing staff/medical technicians play a crucial role in the teaching process when initiating I-Jet pump therapy for PAH patients. What was your experience?
10. On average, how much time was needed to show all I-Jet pump features to first time user-patients (time spent per patient on training)?
11. Did your patients regularly contact you or the training doctor after first use of the I-Jet pump?
12. What was the most frequent issue they struggled with?
13. Was it easy to solve those issues?
14. Do you have any suggestions for other people who train PAH patients on I-Jet with regard to how to initiate therapy and make this successful?
15. What do you think is the best way to teach new users?
16. What are the advantages and disadvantages of employing an I-Jet pump device?
17. How would you rate your overall experience with the I-Jet pump?
18. How would you rate the patient´s satisfactions with the I-Jet pump?
19. How would you rate the devices method of preparation and syringe refill process?
20. How would you rate the safety of the I-Jet pump?
21. Did the patients received initial training on what to do in case of an occlusion alarm?
22. Were the patients equipped with back up pumps in their own homes?
23. Was it necessary to use back up pump during the survey period?
24. Did the patient received initial training on how frequently it is recommended to change infusion site?
25. Is the I-Jet pump infusion pump a reliable self management tool for PAH patients?
26. Would you recommend its use for other PAH patients because you believe it to be a user-friendly device that is easy to learn and provides satisfactory treatment option?
27. Please describe the up-titration period. What was the average flow rate of Remodulin when your patients started I-Jet pump use in 2020?
28. Please describe the up-titration period. On average how much time was needed to reach the treatment goal/maximum dose in new patient.
29. Please describe the infusion site pain management techniques of your patients.
30. How common was site pain in PAH patient users?
31. Please rate your overall satisfaction with the I-Jet pump technical features. Technical performance valuation
    a. Pump size

b. Keypad/menu
c. Display
d. Alarm set up
e. Refill process
f. Occlusion alarm feature
g. Programming speed
h. Noise
i. Reliability-consistancy in performance
j. Alerts messages
k. Pump waterproofing

32. Convenience
    a. Initial set up of device/I-Jet
    b. Maintaining device function
    c. Adjusting device due to dosing rescheduling increase
    d. Educating/reinstructing patients on how to use the device

Supplementary Table S2. Patient training on the use of the I-Jet infusion pump

**Training details**	
Format	Presential training by a trainer nurse in an outpatient visit
Content	**Basic functions of the I-Jet infusion pump:**<br>1. Control buttons<br>2. Insertion of battery/ turning the pump on and off<br>3. Display screens<br>4. Setting date and time<br>5. Syringe and infusion set<br>6. Changing the syringe<br>7. Priming the tubing<br>8. Stopping and restarting the pump<br>9. Infusion rate<br>10. Battery life<br>11. Remaining medication in the syringe<br>12. Lock mode<br>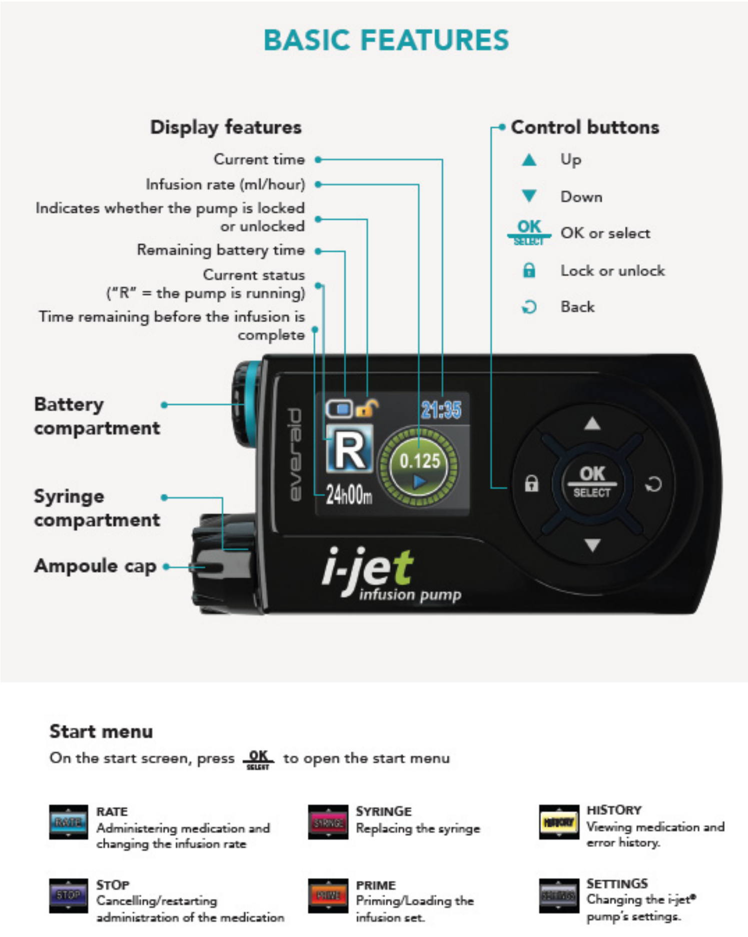<br>**Applied functions and the warnings:**<br>1. How to view the medication delivery history and priming history<br>2. How to manage the settings: date/time, display contrast, prime limit and self-test<br>3. Possible warnings and action to sort out the problem

Warnings

Screen Messages	Alarm & Grade	Cause	Action
WARNING<br>System Error	Grade : HIGH<br>Beep or vibration every second until user press the OK/SELECT button.	System Error	▸ Remove infusion set from your body immediately<br>▸ Contact your health care professional or your service representative immediately.<br>▸ Set up an alternative infusion system as per your health care providers instructions

CAUTION
· Even if the vibrator is switched off, it will be activated for safety reasons when an Error occurs.
· Vibration is disabled when pump has a Low Battery.

Screen Messages	Alarm & Grade	Cause	Action
WARNING<br>Low Battery	Grade : HIGH<br>Beep or vibration every second until user press the OK/SELECT button.	Low Battery Battery with remaining charge of 10% or less.	▸ Press OK/SELECT button to confirm the screen message.<br>▸ Change battery immediately<br>▸ Refer to Page 11.
WARNING<br>Occlusion	Grade : HIGH<br>Beep or vibration every second until user press the OK/SELECT button.	No delivery - Occlusion	▸ Disconnect the infusion set immediately from your body (Detach the tubing from the needle/catheter or remove the needle /catheter) and check the following.<br>▸ Check for occlusion of the infusion set. Visually check for any possible occlusion of the Infusion Set and take corrective measures if available (e.g. pinch, direct pressure, etc.). If this does not resolve the problem change the Infusion Set.
WARNING<br>Empty Syringe	Grade : HIGH<br>Beep or vibration every second until user press the OK/SELECT button.	No remaining medication in the syringe	▸ Check if the medication Syringe is empty. Visually confirm remaining medication through the Syringe cavity window on the side of the pump. After confirming that the Syringe is empty put a new Syringe of medication in the pump following the instructions on page 15 - 16.

CAUTION
· For safe and sure delivery, it is recommended that you change the Syringe when 0.1 ml medication remains.

WARNING
· If the "warning" message continues to appear after taking all of the above measures stop using the pump. Remove the battery and contact your service representative immediately.

Screen Messages	Alarm & Grade	Cause	Action
WARNING<br>Low Volume<br>WARNING<br>Low Volume	Grade : LOW<br>Beep or vibration every second until user press the OK/SELECT button.	When 3hours of medication remain	▸ Press the OK/SELECT button to confirm the screen message.<br>▸ Refer to Page 20

CAUTION
· For safe and sure delivery, it is recommended that you change the Syringe when there are any of the following volume alarms:

Maintenance of the I-Jet pump:

1. Cleaning
2. How to clean the pump
3. Pump storage
4. Specific precautions

Preparation of the pump for the first use:

Step 1 and 2: Insert the subcutaneous needle and program the I-Jet pump

1<br>INSERT SUBCUTANEOUS NEEDLE	2<br>PROGRAM THE I-JET PUMP

NB! If the existing needle is to be used, go straight to point 2.

A. Take out the subcutaneous needle and hold up the front edge of the tape.

B. Remove the blue needle cover from the needle.

C. Insert the needle at a 30-45 degree angle. Penetrate the skin with a swift and firm motion ensuring that the needle goes all the way in.

D. Remove the protective backing from the front section of the tape and attach the tape to the skin.

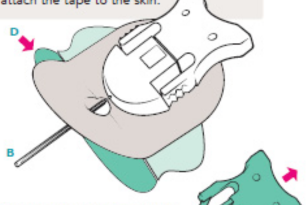

E. Remove the metal needle by pressing the serrated part in the middle.

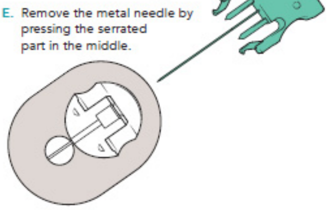

F. Remove the protective backing from the back section of the tape and attach it to the skin.

A. Insert the battery.

B. If necessary, unlock the pump by pressing the lock button and the down arrow for 5–7 seconds.

C. Press [OK/SELECT], and toggle up/down until [RATE] is displayed in blue.

D. Press [OK/SELECT] again.

E. Use the [UP/DOWN BUTTONS] until the prescribed infusion rate is displayed.

F. Press [OK/SELECT] to confirm.

G. The programmed rate is now displayed in the circle.

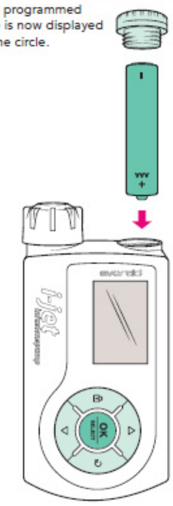

Step 3 and 4: load the medication in the syringe and prepare connections

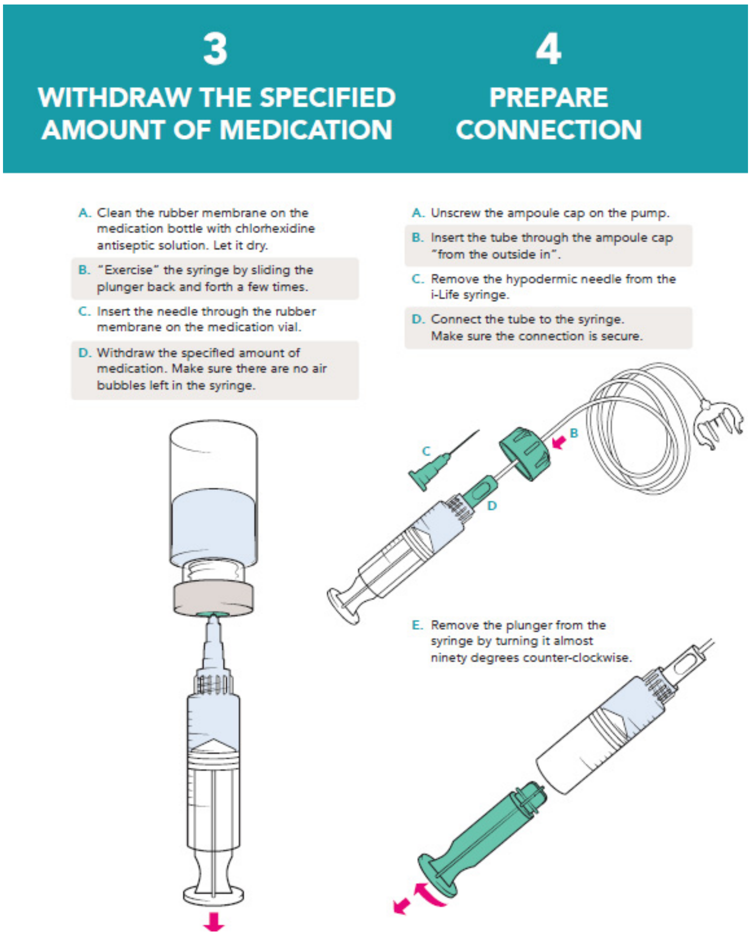

Step 5, 6 and 7: connect the syringe to the pump and the subcutaneous needle

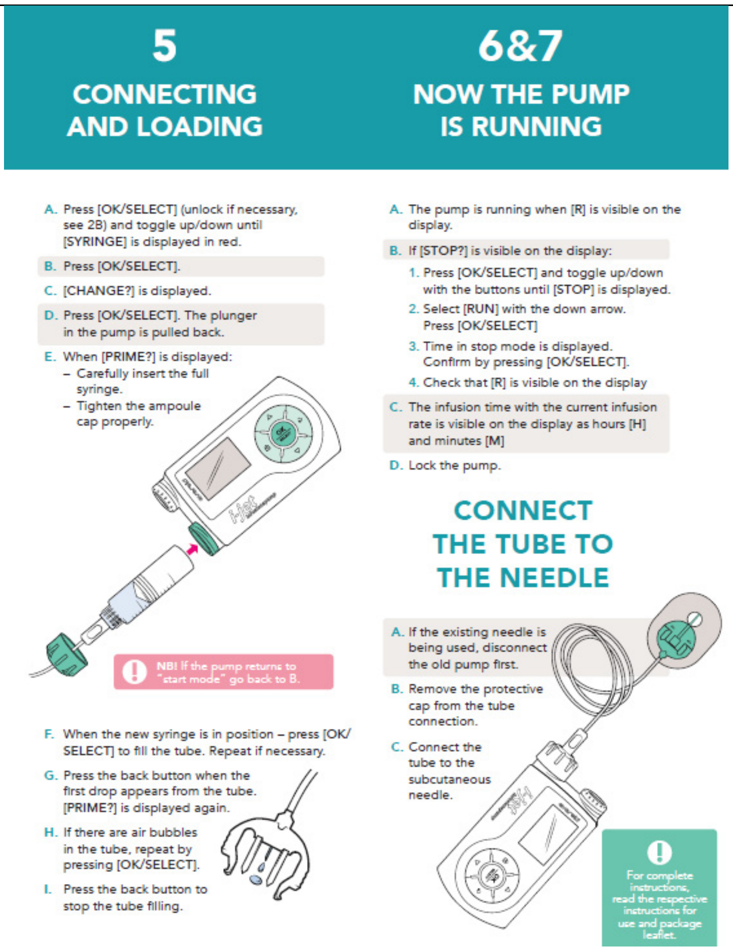

Frequency	One training
Duration	2-3 hours
Setting	John Paul II Hospital, Krakow, Poland
**Support material** (support material was given to the patients to take them home)	
• User manual<br>• Leaflet<br>• Video	
**Follow up**	
• 4 weeks after training: phone call<br>• 8 weeks after training: outpatient visit to adjust treatment if necessary<br>• Patients can contact the nurse during the study at any time if necessary	
